# Photosynthetic Properties and Potentials for Improvement of Photosynthesis in Pale Green Leaf Rice under High Light Conditions

**DOI:** 10.3389/fpls.2017.01082

**Published:** 2017-06-20

**Authors:** Junfei Gu, Zhenxiang Zhou, Zhikang Li, Ying Chen, Zhiqin Wang, Hao Zhang, Jianchang Yang

**Affiliations:** Jiangsu Key Laboratory of Crop Genetics and Physiology/Co-Innovation Center for Modern Production Technology of Grain Crops, Yangzhou UniversityYangzhou, China

**Keywords:** chlorophyll, electron transport rate, light-harvesting chlorophyll antenna, non-photochemical quenching, reactive oxygen species, photosynthesis

## Abstract

Light is the driving force of plant growth, providing the energy required for photosynthesis. However, photosynthesis is also vulnerable to light-induced damage caused by the production of reactive oxygen species (ROS). Plants have therefore evolved various protective mechanisms such as non-photochemical quenching (*NPQ*) to dissipate excessively absorbed solar energy as heat; however, photoinhibition and *NPQ* represent a significant loss in solar energy and photosynthetic efficiency, which lowers the yield potential in crops. To estimate light capture and light energy conversion in rice, a genotype with pale green leaves (*pgl*) and a normally pigmented control (Z802) were subjected to high (HL) and low light (LL). Chlorophyll content, light absorption, chloroplast micrographs, abundance of light-harvesting complex (LHC) binding proteins, electron transport rates (ETR), photochemical and non-photochemical quenching, and generation of ROS were subsequently examined. *Pgl* had a smaller size of light-harvesting chlorophyll antenna and absorbed less photons than Z802. *NPQ* and the generation of ROS were also low, while photosystem II efficiency and ETR were high, resulting in improved photosynthesis and less photoinhibition in *pgl* than Z802. Chlorophyll synthesis and solar conversion efficiency were higher in *pgl* under HL compared to LL treatment, while Z802 showed an opposite trend due to the high level of photoinhibition under HL. In Z802, excessive absorption of solar energy not only increased the generation of ROS and *NPQ*, but also exacerbated the effects of increases in temperature, causing midday depression in photosynthesis. These results suggest that photosynthesis and yield potential in rice could be enhanced by truncated light-harvesting chlorophyll antenna size.

## Introduction

Global agriculture is facing unprecedented challenges, with a predicted increase in primary foodstuffs of 85% required by 2050, relative to 2013 (Ray et al., 2013). In addition, yield improvements are beginning to slow or stagnate with the Green Revolution reaching its biological limits. Accordingly, photosynthesis, which has shown few improvements in crop breeding, remains the key to increased genetic yield potential (Long et al., 2015). Reducing the energy losses during photosynthesis could help improve solar energy conversion efficiency, thereby boosting crop yields (Gu et al., 2014; Furbank et al., 2015; Yin and Struik, 2015).

Non-photochemical quenching (NPQ) is a major cause of solar energy conversion efficiency loss, not least because C_3_ leaf photosynthesis is saturated at ~25% maximum sunlight (Long et al., 2006). The energy of irradiated light is therefore often much higher than the demand for photosynthetic metabolism of NADPH and ATP (Melis, 1999; Niyogi, 2000; Miyake et al., 2005; Yamori and Shikanai, 2016). Excess excitation of plant photosystems results in over excitation of the reaction center, causing chlorophyll molecules to attain a triple state, ^3^P680^*^ (Hideg et al., 1998). ^3^P680^*^ reacts rapidly with O_2_, resulting in production of deleterious singlet O_2_ (^1^O_2_) (Vass and Cser, 2009), which oxidatively degrades D_1_ protein, a PSII reaction center protein, inactivating PSII (Krieger-Liszkay et al., 2008). The PSII reaction center is so vulnerable to light-induced damage that it has to be rebuilt approximately once every 30 min (Foyer and Shigeoka, 2011). Furthermore, inherent limitations in the capacity for electron transport through the cytochrome *b*_6_/*f* complex lead to the production of photoexcited Chl, ^1^Chl^*^, and its triplet state, ^3^Chl^*^, causing ^1^O_2_ accumulation in the thylakoid membranes (Macpherson et al., 1993; Telfer, 2014), which stimulates peroxidation and degradation of membrane bilayers. To protect PSII from excess radiation, plants dissipate excessive energy as heat via the xanthophyll cycle, which involves de-epoxidase-induced catalysis of the xanthophyll pigment violaxanthin to zeaxanthin. This photoprotection process is referred to as *NPQ* of chlorophyll fluorescence (Niyogi et al., 1998; Miyake et al., 2005; Yamori and Shikanai, 2016), and represents a significant loss of solar energy (Ort et al., 2015).

During the photosynthetic process, a minimum of eight photons are required to assimilate one molecule of CO_2_: (i) assimilation of 1 mol CO_2_ in the Calvin-Benson cycle requires 2 mol NADPH; (ii) reduction of NADP^+^ to NADPH involves the transfer of 2 electrons; and (iii) movement of 1 mol of electron along the linear electron transport chain through PSII and PSI requires 1 mol photon absorption by each photosystem. Moreover, taking into account the spectrum of sunlight that is used for photosynthesis as well as the absorption efficiency of the leaves, the energy in natural solar radiation and the amount of CH_2_O in glucose, the maximum solar energy conversion efficiency can reach up to 12.3% (Yin and Struik, 2015). However, in annual crops, the typical solar energy conversion efficiency is very low, usually < 1% (de Groot, 2008). Thus, huge energy losses occur in plant photosystems compared to the theoretical maximum with actual solar energy conversion efficiency, in which the fraction of energy loss due to *NPQ* increases with increasing solar radiation, reaching up to 60% of captured sunlight under full sunlight (Yin and Struik, 2015). One way to increase solar conversion efficiency and reduce *NPQ* is to reduce antenna size of the photosystems. If too large, the antennae have been shown to trap more light than can be used. Thus, if plants had fewer light-harvesting pigments (e.g., chlorophyll and carotenoids) per photosystem, solar energy conversion efficiency could be greatly improved (Melis, 2009; Ort et al., 2011, 2015; Long et al., 2015). This suggests that there is potential to reduce the size of chlorophyll antennae, thereby lowering energy waste through NPQ and improving solar energy conversion efficiency.

In the crop canopy, reducing the chlorophyll content would not only mitigate efficiency losses associated with NPQ but also allow greater transmittance of light into lower layers, thus improving canopy light distribution and canopy photosynthesis (Pettigrew et al., 1989; Ort et al., 2011; Gu et al., 2017). In green alga, the ***tla*** mutant was found to have improved photosynthetic solar energy conversion efficiency and productivity by up to three-fold compared to the wild-type due to the truncated chlorophyll antenna size of its photosystems (Melis et al., 1998; Polle et al., 2003; Melis, 2009). In higher plants, decreased leaf chlorophyll content has also been shown to be advantageous in terms of photosynthetic efficiency in rice (Gu et al., 2017) and soybean (Pettigrew et al., 1989). A decrease in leaf chlorophyll content could also be evolutionarily advantageous in high light and high temperature environments (Tardy et al., 1998), since lowering leaf chlorophyll content has also been shown to act as a photoprotection mechanism, mitigating the damaging effects of high radiation and high leaf temperature in wild grasses and cereal landraces adapted to semi-arid environments (Havaux and Tardy, 1999; Zaharieva et al., 2001; Royo et al., 2014). Under long-term acclimation to high light environments, plants adjust to the environment by increasing antioxidant production and decreasing light harvesting antenna size through regulated gene expression (Foyer and Noctor, 2009). However, in rice, it is unknown whether and to what extent photo-oxidative stress is relieved and solar energy conversion efficiency improved via selection of a genotype with reduced chlorophyll content. Accordingly, the benefits at the canopy level also remain unknown.

Previous research aimed at improving photosynthesis has focused mainly on optimization of the Calvin cycle, which assimilates and reduces carbon dioxide conversion to carbohydrates. Approaches have included designing more efficient Rubisco, increasing mesophyll conductance, introducing a CO_2_-concentrating mechanism in C_3_ crops, and short-circuiting photorespiration (Mueller-Cajar and Whitney, 2008; Uehlein et al., 2008; Whitney and Sharwood, 2008; Maurino and Peterhansel, 2010; von Caemmerer et al., 2012). However, less attention has been paid to optimization of light capture and solar energy conversion during light reactions (Ort et al., 2015). In this study, we used the early *indica* rice variety Zhefu 802 and a pale green leaf genotype originating from Zhefu 802 to examine the hypothesis that truncated light-harvesting chlorophyll antenna size could improve photosynthesis in rice. Chlorophyll content, light absorption, chloroplast development, electron transport, photochemical and non-photochemical quenching, and generation of ROS were examined in both genotypes to (i) investigate photosynthetic efficiency in the chlorophyll-deficient and normal genotype; (ii) examine the effects of high light intensity on oxidative stress in the two genotypes; and (iii) determine the relationship between chlorophyll content and diurnal variation in photosynthesis under field conditions. The results would provide valuable insight into the mechanisms of photoprotection and increased photosynthetic efficiency with truncated light-harvesting chlorophyll antenna size, helping development of strategies for improved grain yield potential in rice.

## Materials and methods

### Plant material and growth conditions

The *pgl* genotype with pale green leaves was generated by radiation from the early *indica* rice variety Zhefu 802 (Z802). In this study, both the *pgl* and Z802 genotype were used to examine the effects of chlorophyll concentration on photosynthetic characteristics.

Experiments were conducted at the research farm of Yangzhou University, Jiangsu Province, China (32°30′ N, 119°30′ E). The soil was sandy loam (Typic Fluvaquent, Etisol) with 23.2 g kg^−1^ organic matter, 95.2 mg kg^−1^ alkali-hydrolyzable *N*, 22.5 mg kg^−1^ Olsen-P, and 82.6 mg kg^−1^ exchangeable K. Seeds were first sown in the paddy field on 12 May 2015.

In the pot experiment, seedlings were transplanted to pots (30 cm in height and 25 cm in diameter, 14.7 L in volume) on 13 June 2015. Each pot was filled with 20 kg of sandy loam soil, three hills per pot and two seedlings per hill. On the day of transplanting, 0.5 g of KH_2_PO_4_ was mixed with the soil in each pot as basal fertilizer. The nitrogen rate was 3.6 g urea per pot. The urea was applied at pre-transplanting (1 day before transplanting), early tillering (~7 days after transplanting), and the panicle initiation stage (40 days after transplanting) at rates of 50, 20, and 30%, respectively. At the 5th leaf stage, plants were moved to climate chambers under the following photosynthetic photon flux densities (PPFD): ~150 μmol m^−2^ s^−1^ (low light, LL) and 1,000 μmol m^−2^ s^−1^ (high light, HL) at the canopy level during the light period. All other environmental factors were kept the same. The temperature in the climate chambers was set at 28°C for a 12 h light period and 23°C for a 12 h dark period. CO_2_ was set at 380 μmol mol^−1^ and the relative humidity at 65%. The pots were kept in the climate chambers for 20 days up until expansion of the 7th leaf. The 7th leaf was then sampled for physiological analysis, having fully acclimated to the relative light environment.

In the field experiment, seedlings were transplanted to the field on 13 June 2015 at a hill spacing of 25 × 15 cm with two seedlings per hill. P (30 kg ha^−1^ as single superphosphate) and K (40 kg ha^−1^ as KCl) were applied and incorporated just before transplanting as basal fertilizer. Three levels nitrogen (0, 120, or 240 kg *N* ha^−1^) in the form of urea was applied. The time and proportion of urea application were the same as in the pot experiment. The experiment followed a split-plot design with three replicates per treatment, using nitrogen as the main plot and genotype as the sub-plot variable. Each plot was 7 × 6 m. Weeds were controlled by a combination of chemical and manual methods, and insects were controlled chemically.

### Leaf optical measurements

Leaf optical measurements were conducted using a double integrating sphere system. An ISP-REF integrating sphere (Ocean Optics, Inc., Dunedin, Florida, USA) with a built-in collimated light source and a gloss-trap combined with a FOIS-1 fiber optic integrating sphere (Ocean Optics) were used. Both spheres were connected via fiber optic bundles to a S2000 UV-VIS-shortwave NIR spectroradiometer (Ocean Optics) with a resolution of ~0.5 nm. They were aligned using a rack-and-pinion slide (NT61–285, Edmund Industrial Optics, Barrington, New Jersey, USA) with a platform for each sphere. By clamping a leaf between the two integrating spheres, simultaneous measurements of leaf spectral reflectance (R) and transmittance (T) were possible. Absorptance (A) was calculated as A = 1 − (R+T).

### Electron microscopy

Leaf sections ~1–2 mm^2^ were cut from the middle of newly expanded leaves using two razor blades, fixed in 2.5% glutaraldehyde (0.1 mol l^−1^ phosphate buffer, pH 7.4) then post-fixed with 2% osmium tetroxide. Specimens were dehydrated in a graded acetone series and embedded in Epon 812. Samples were then dissected using a Power Tome-XL ultramicrotome, stained with 2% uranyl acetate and examined with a CM 100 transmission electron microscope (Philips, Netherlands).

### Rapid light curve measurements

Fluorescence measurements were performed using a MINI-PAM-II fluorometer (Walz, Germany). Rapid light curves (RPCs) were initiated at light intensities of 26, 46, 67, 94, 131, 199, 298, 441, 661, 860, 1,203, and 1,568 μmol m^−2^ s^−1^, at a duration of 30 s illumination per light intensity. At each light level, fluoresce signal *F* and maximum fluorescence after a saturating light pulse *F*′_m_ were recorded, and the effective quantum yield of PSII (Φ_PSII_), photochemical quenching (*qP*), and non-photochemical quenching (*NPQ*) calculated as follows (Genty et al., 1989; Bilger and Björkman, 1990; Ralph and Gademann, 2005):

(1) $$\Phi_{\text{PSII}}=\Delta F/{F^{\prime }}_{m}=({F^{\prime }}_{m}-F)/{F^{\prime }}_{m}$$

(2) $$qP=({F^{\prime }}_{m}-F)/({F^{\prime }}_{m}-F_{o})$$

(3) $$NPQ=(F_{m}-{F^{\prime }}_{m})/{F^{\prime }}_{m}$$

Changes in the electron transport rate (ETR; μmol electron m^−2^ s^−1^) with increasing photosynthetic active radiation (PAR, 0–1,568 μmol m^−2^ s^−1^) were used to generate the RLCs. Relative ETR (*r*ETR) represents a relative measurement of electron transport through the photochemical reactions leading to carbon fixation:

(4) $$r\text{ETR}=\text{PAR}\times \Phi_{\text{PSII}}\times 0.5\times \chi$$

where 0.5 is a multiplication factor of two quanta of light required for the transport of one electron, and χ is the fraction of incident quanta absorbed by leaves from genotype Z802 or *pgl*.

Photosynthetic parameters were estimated from the RLCs using the equation of Platt et al. (1980):

(5) $$P=P_{s}\;[1-\text{exp}(-\alpha E/P_{s})]\text{ exp}(-\beta E/P_{s})$$

In the absence of photoinhibition (β = *0*), the function becomes a standard rectangular hyperbola, with an asymptotic maximum *r*ETR value (Harrison and Platt, 1986). The following simplified equation can be applied:

(6) $$P=P_{m}[1-\text{exp }(-\alpha E/P_{m})]$$

where *P*_s_ is a scaling parameter defined as the maximum potential *r*ETR, *P*_m_ is the photosynthetic capacity at saturating light, α is the photosynthetic efficiency measured by the initial slope of the RLC before the onset of saturation, *E* is the light density, and β characterizes the slope of the RLC where PSII declines (Henley, 1993). *r*ETR_max_ (relative electron transport maximum) and *E*_*k*_ (minimum saturating irradiance) were estimated as:

(7) $$r{\text{ETR}}_{\text{max}}=P_{s}[\alpha /(\alpha +\beta )]{\left[\beta /(\alpha +\beta )\right]}^{\beta /\alpha }$$

(8) $$\begin{array}{c} E_{k}={\mathrm{ETR}}_{\text{max}}/\alpha \end{array}$$

For curve fitting, iterative non-linear least square regression was carried out using the GAUSS method in PROC NLIN of SAS (SAS Institute Inc., Cary, NC, USA).

### Quantitative analysis of protein by the isobaric tags for relative and absolute quantification (iTRAQ)

The procedure of quantitative proteomic analysis was essentially identical as described previously (Yu et al., 2016), including the steps of protein extraction, in solution and iTRAQ labeling, strong cation exchange, and LC-MS/MS. For protein identification, it was performed by using Mascot search engine (Matrix Science, London, UK; version 2.3.02) against database containing rice sequences. The charge states of peptides were set to +2 and +3. Specifically, an automatic decoy database search was performed in Mascot by choosing the decoy checkbox in which a random sequence of database is generated and tested for raw spectra as well as the real database. To reduce the probability of false peptide identification, only peptides at the 95% confidence interval by a Mascot probability analysis greater than “identity” were counted as identified. And each confident protein identification involves at least one unique peptide. For protein quantitation, it was required that a protein contains at least two unique peptides. The quantitative protein ratios were weighted and normalized by the median ratio in Mascot. Therefore, the proteins with *p* < 0.05, and fold-change ratios ≥1.5 or ≤ 0.67 were considered as significant.

### ROS detection by fluorescence microscopy

Superoxide radicals were detected by staining with 10 μM dihydroethidium (DHE) (Yamamoto et al., 2002). As a negative control, transections of leaves were incubated with 1 mM N_3_Na (peroxidase inhibitor). Fluorescent images were captured using an Axio Imager D2 microscope (Zeiss, Oberkochen, Germany) with DHE fluoresces red (540 nm excitation, 590 nm emission).

### Analysis of lipid peroxidation and antioxidant enzyme activity

The content of malondialdehyde (MDA), a sign of lipid peroxidation, was estimated by measuring the concentration of thiobarbituric acid reactive substances (TBARS) (Dhindsa et al., 1981). A superoxide dismutase (SOD) activity assay was conducted based on the inhibition of the photoreduction of nitroblue tetrazolium (NBT) as described by Giannopolitis and Ries (1977). One unit of SOD activity was defined as the amount of enzyme causing 50% inhibition of the reaction in the absence of an enzyme under these conditions. A peroxidase (POD) activity assay was conducted based on the conversion of guaiacol to tetraguaiacol monitored at 470 nm, as described by Chance and Maehly (1955). Catalase (CAT) activity was measured according to the method of Aebi (1983) with some modifications. The CAT reaction mixture contained 25 mM sodium phosphate buffer (pH 7.0) and 40 mM H_2_O_2_. The reaction was initiated by adding enzyme extract, and the change in absorbance of the reaction solution at 240 nm recorded every 20 s.

### Thermal imaging of the rice canopy

The temperature of leaves in the rice canopy was determined using an FLIR ThermaCAM™ S65 system (FLIR Systems Inc., Portland, OR. USA) with a wide-angle camera lens (18 mm IR-LENS), the spectral and detectable temperature ranges of which are 7.5–13 mm and −40 to 1,500°C, respectively. Temperature differences of <0.06°C (30°C, 50 Hz) can be detected. Data were analyzed using Therma CAM Researcher Pro 2.7 software (FLIR Systems Inc., Portland, USA).

### Photosynthesis measurements

For light-saturated photosynthesis, the middle parts of fully expanded leaves were measured using a portable open gas exchange system (Li-6400, Li-COR Inc., Lincoln, NE, USA) with a 2 × 3 cm leaf chamber (LI-6400-02 LED, Li-COR Inc., Lincoln, NE, USA) at an ambient CO_2_ concentration, similar to Cui et al. (2016), but with light intensity set at 1,500 μmol m^−2^ s^−1^, leaf temperature set at 25°C. For measurements of diurnal variation of the net photosynthesis, measurements were taken on clear days around flowering stage with natural fluctuation of air temperature and vapor pressure deficit. The PPFD used for each assessment was provided by the artificial light source (LI-6400-02 LED, Li-COR Inc., Lincoln, NE, USA), which was set to provide the same PPFD as solar intensity that was tracked by an external light sensor of LI-6400 near leaf chamber.

## Results

### Chlorophyll content, biomass, photosynthesis, anatomical structure and the effects on light absorption

**Figure 1:**
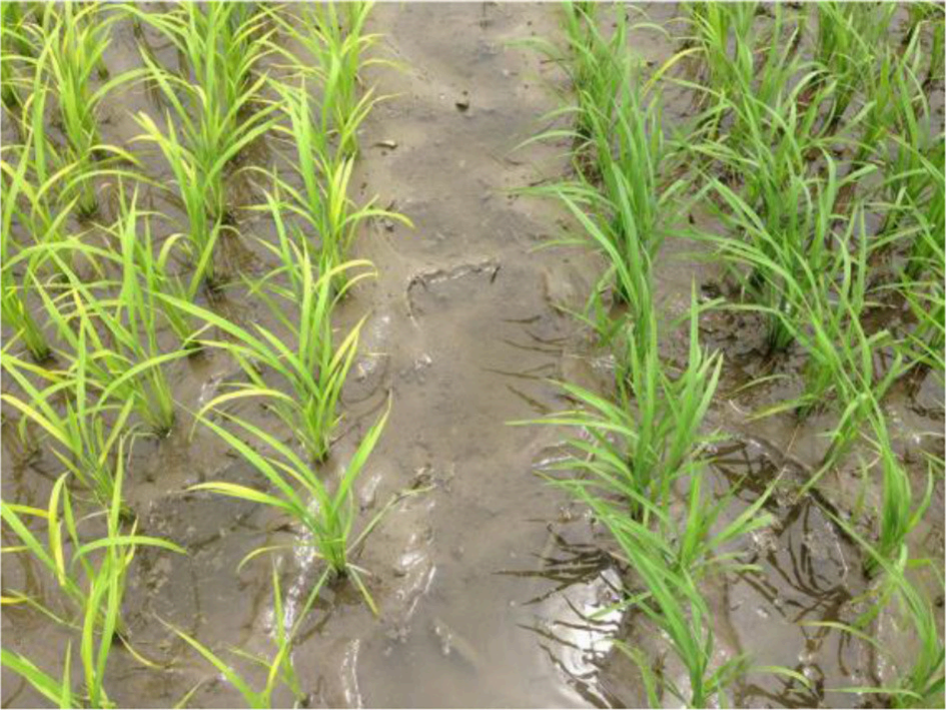
Genotype Z802 **(right)** and *pgl* **(left)** at the seedling stage.

**Figure 2:**
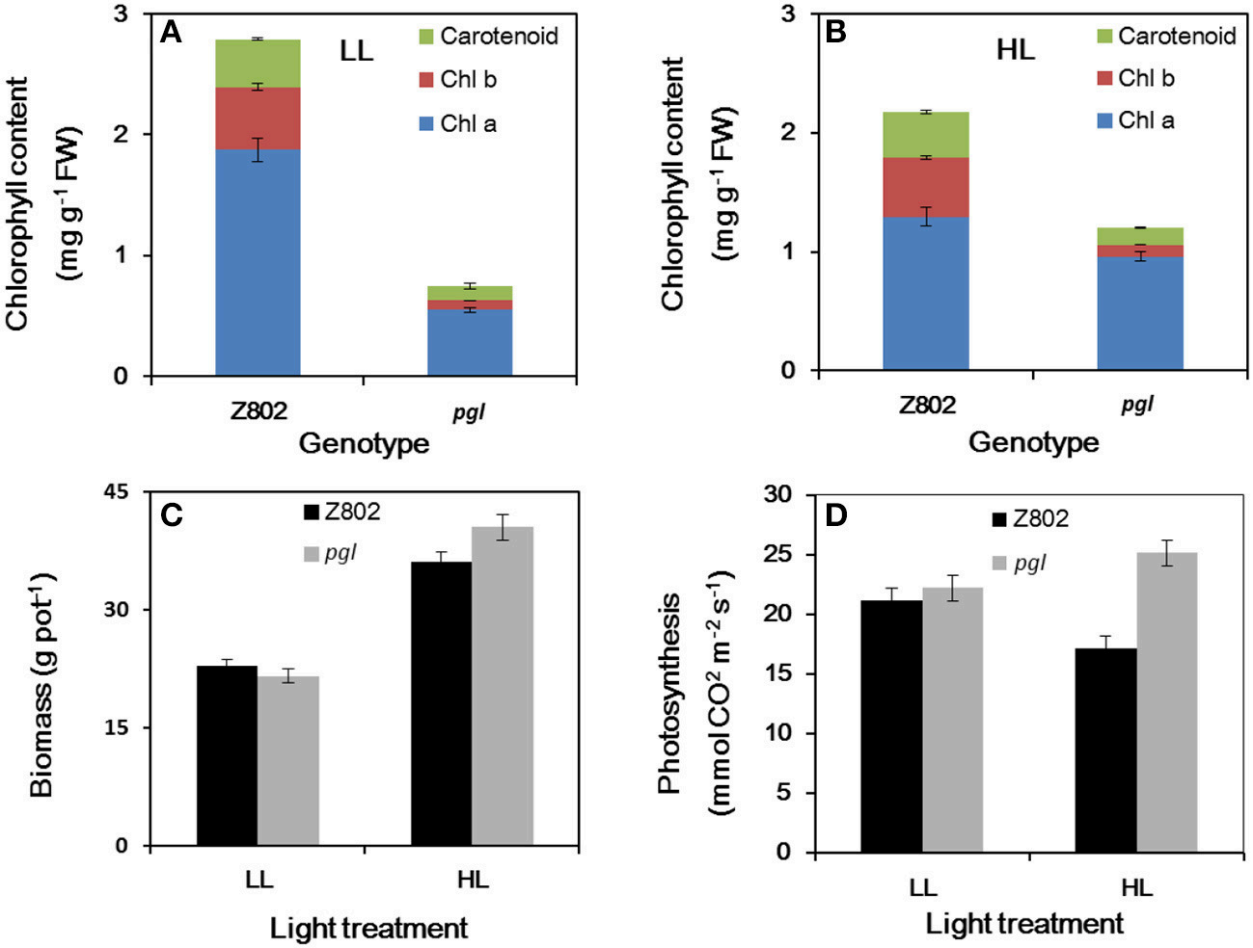
Chlorophyll content **(A,B)**, biomass **(C)**, light-saturated photosynthesis **(D)** for Z802 and *pgl* from high (HL) and low light (LL) adapted plants. Data represent the means of three replicates per treatment ± standard deviation. The photosynthesis was measured at light intensity of 1,500 μmol m^−2^ s^−1^, temperature of 25°C with Li-6400.

The pale green leaves of the *pgl* genotype (Figure 1) were consistent with its significantly lower chlorophyll content (0.75 mg g^−1^ FW under LL and 1.20 mg g^−1^ FW under HL; Figure 2) compared to Z802 (2.79 and 2.18 mg g^−1^, respectively). Under LL and HL, the chlorophyll content of *pgl* was 27 and 55% that of Z802, respectively. Compared with LL treatment, the chlorophyll content of Z802 decreased by 22% under HL. In contrast, an increase of 60% was observed in *pgl*. The ratio of chlorophyll *a* to *b* (Chl *a*/*b*) were greatly improved from 3.6 to 7.0 and 2.6 to 9.6 for LL and HL treatments, respectively, when compare chlorophyll in Z802 with in *pgl* (Figure 2).

There were not significant differences in biomass between two genotypes in LL treatment, while in HL treatment, the biomass of *pgl* is significantly higher than Z802 (Figure 2). The light-saturated photosynthesis is much higher in *pgl* than in Z802 in HL treatment. When compared LL with HL treatments, the light-saturated photosynthesis is decreased for Z802, but increased for *pgl* (Figure 2). Negative correlations between chlorophyll content and biomass and light-saturated photosynthesis could be found in Figure 2, especially for the HL treatment. For the field experiment, the yield, yield components, and biomass of Z802 and *pgl* in the field experiment also indicate a potential for production improvements by a smaller antenna size (Table S1).

**Figure 3:**
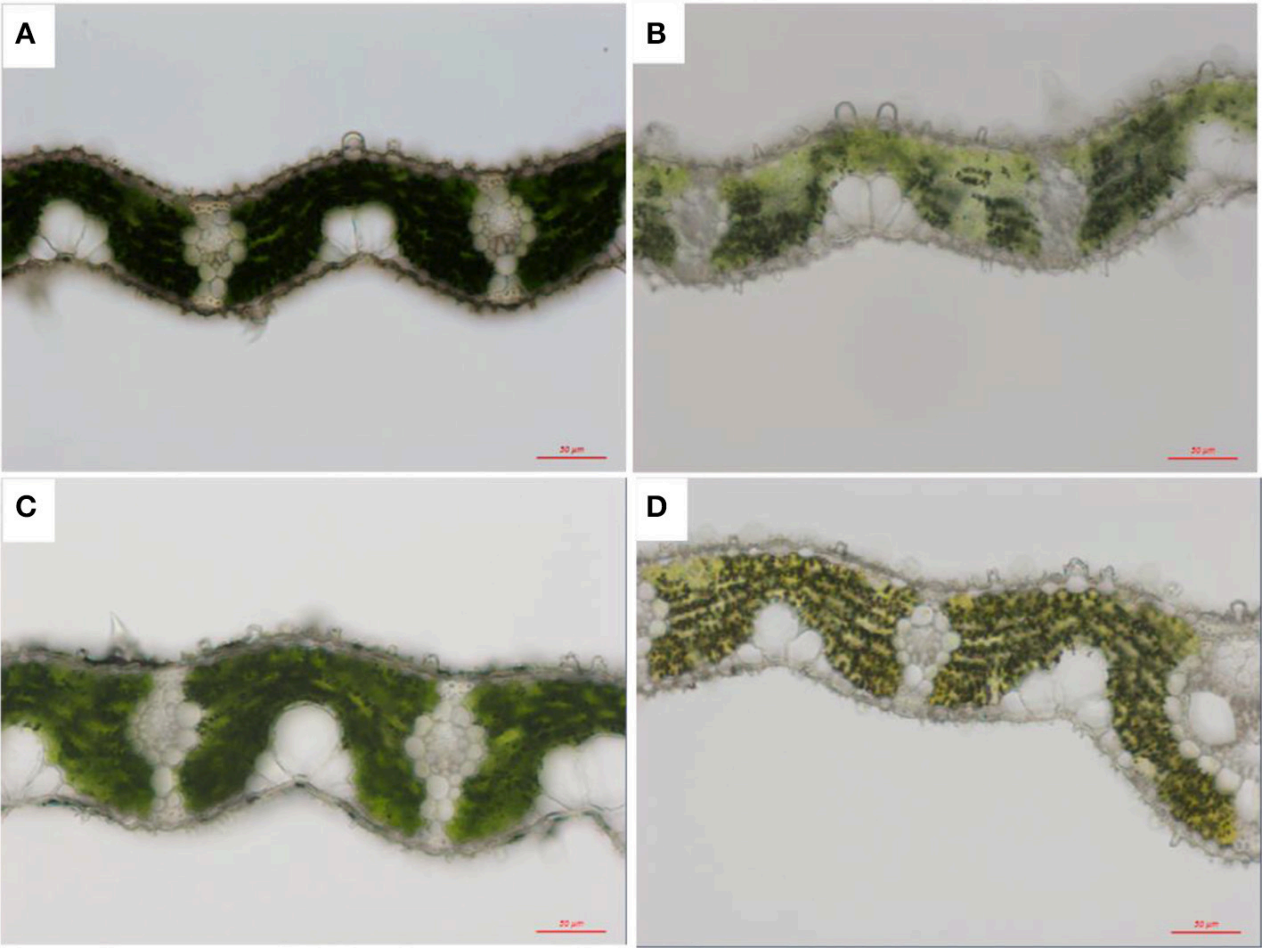
Light micrographs of chloroplasts under low (LL; **A,B**) and high light treatment (HL; **C,D**) in Z802 **(A,C)** and *pgl* **(B,D)**. Bars = 50 μm.

Anatomical structure was also influenced by light (Figure 3). Leaf thickness was greatly improved with increasing light intensity in both genotypes from 87.87 ± 7.51 to 112.37 ± 9.24 μm in Z802 under LL and HL, respectively, and from 82.65 ± 4.0 to 110.3 ± 8.12 μm in *pgl*, respectively. Increasing leaf thickness also resulted in a corresponding increase in leaf dry matter per unit area (LMA) from 30.5 ± 2.8 under LL to 36.9 ± 2.7 g m^−2^ under HL in Z802, and from 25.2 ± 1.0 to 32.0 ± 2.4 g m^−2^ in *pgl*, respectively.

**Figure 4:**
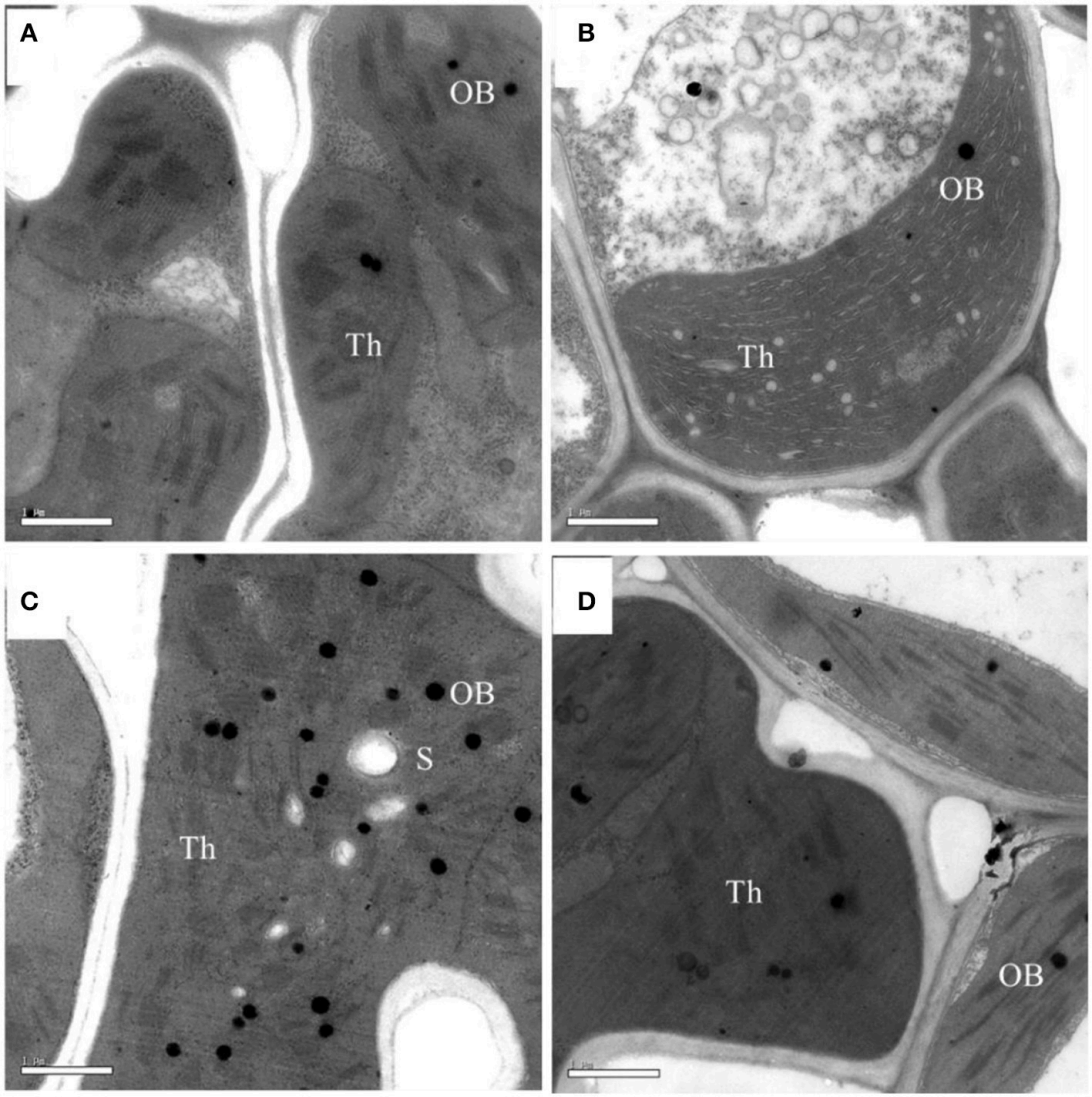
Electron micrographs of chloroplasts under low (LL; **A,B**) and high light treatment (HL; **C,D**) in Z802 **(A,C)** and *pgl* **(B,D)**. Bars = 1 μm. Th, thylakoid lamellae; OB, osmophilic body; S, starch.

Although, the chlorophyll content was significantly lower in the *pgl* genotype, the chloroplasts and grana were normal in both genotypes in terms of size and grana stacking (Figure 4). Comparisons of the chlorophyll micrographs revealed more osmophilic bodies in Z802 than *pgl*, especially under HL. This suggests disruption of the granum thylakoids in Z802 and a possible effect on chloroplast function due to photoinhibitory damage under HL.

**Figure 5:**
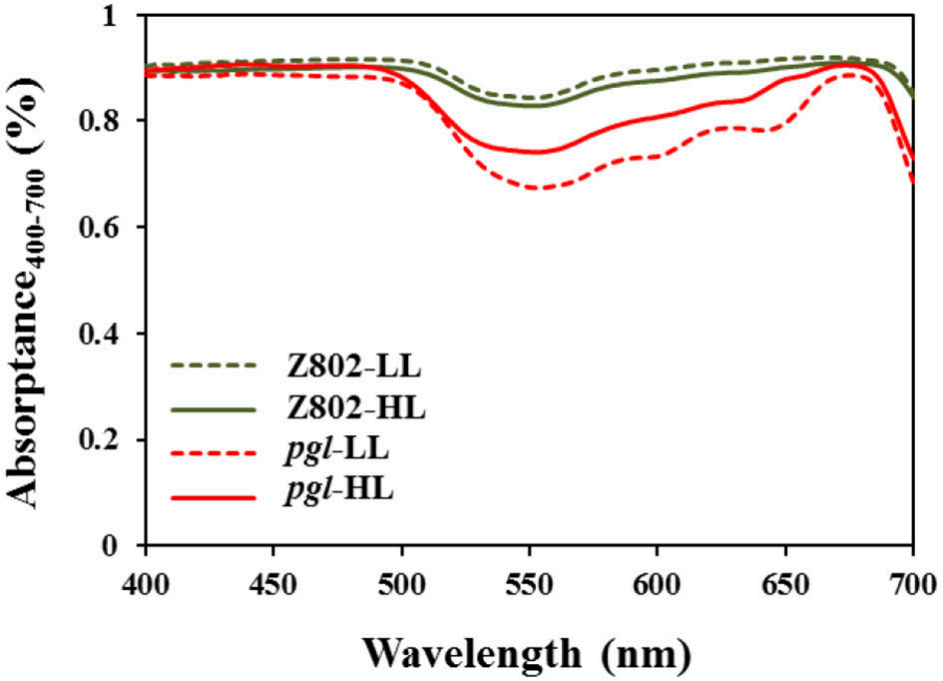
Leaf spectral absorptance rates of leaves of Z802 and *pgl* under low (LL) and high light treatment (HL).

Compared with the significant differences in leaf chlorophyll content (Figure 2), the differences in leaf spectral absorptance rates were comparatively small (Figure 5). In Z802, leaf spectral absorptance was comparable between LL and HL treatment, with leaves adapted to LL absorbing more photons than those adapted to HL. In contrast, an opposite trend was observed in *pgl*. These differences in light absorption might be due to the changes in chlorophyll content shown in Figure 2. For genotype Z802, the absorption rates were 0.898 and 0.883 at LL and HL treatments, respectively. For genotype *pgl*, the absorption rates were 0.808 and 0.846 at LL and HL treatments, respectively.

### Electron transport rates and the solar energy conversion efficiency

**Figure 6:**
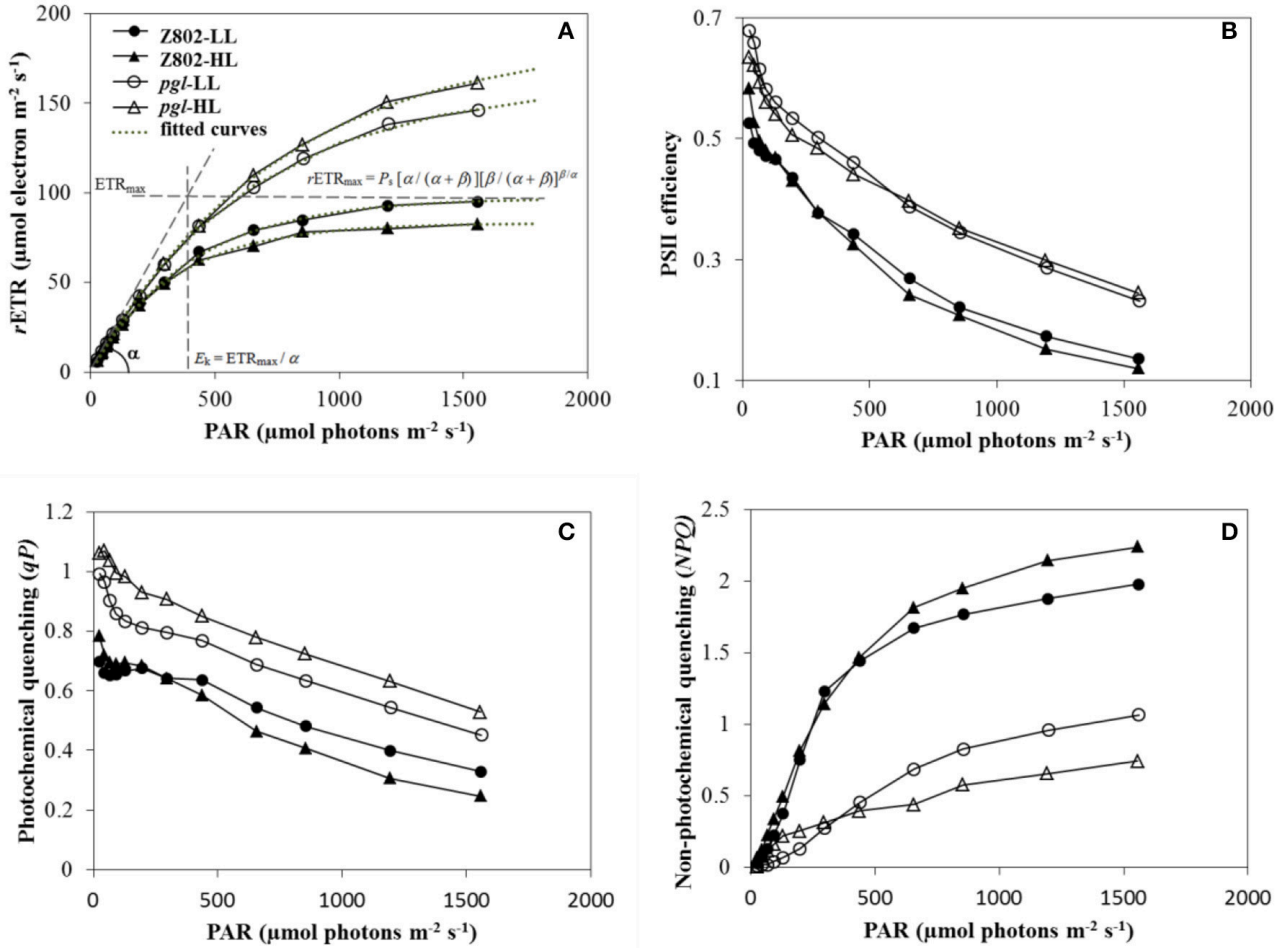
**(A)** Rapid light curves (RLC) of Z802 and *pgl* under high (HL) and low light treatment (LL). The relative electron transport rate (*r*ETR) is plotted against PAR irradiance (μmol photons m^−2^ s^−1^). Fitted curves are plotted with a dotted line, and the *r*ETR_max_, *E*_*k*_, and α are displayed in the RLCs of Z802 under LL. **(B)** PSII efficiency (Φ_PSII_), **(C)** photochemical quenching (*qP*), and **(D)** non-photochemical quenching (*NPQ*) derived from the RLCs under high (HL) and low light (LL) in Z802 and *pgl* as a function of PAR.

Figure 6A indicates the photosynthetic electron transport rates of Z802 and *pgl* under different actinic light with HL and LL treatment. The curves showed a linear increase under limited light followed by a plateau when the photosynthetic pathway became saturated. The average *r*ETR of the *pgl* genotype across different light intensities was 42% higher than that of Z802, with the two genotypes responding differently to light. Leaves of Z802 had a higher *r*ETR under LL than HL, with an opposite tendency in *pgl*. In Figure 6A, fitted curves are plotted with a dotted line, showing very close agreement with the actual data points (*R*^2^ > 99%). Estimated *r*ETR_max_, *E*_*k*_, and α values are also given in the RLCs of Z802 under LL. Since there was no apparent down-regulation of the RLCs, β was set at 0 in the curve fitting. The resulting parameters estimated from curve-fitting are listed in Table 1. The maximum electron transport *r*ETR_max_ of *pgl* was 1.8 times that of Z802 as was the average *E*_*k*_ at 687.0 μmol photons m^−2^ s^−1^. The substantially higher *r*ETR_max_ and *E*_*k*_ values suggest that the *pgl* genotype is able to maintain a higher photosynthetic electron transport capacity. Furthermore, the photosynthetic capacity of *pgl* improved under HL, unlike Z802, which performed better under LL.

**Table 1:** Estimated parameters from rapid light curves for Z802 and *pgl* under low (LL) and high light (HL) by curve fitting.

**Genotype-treatment**	**α**	***r*ETR_max_ (μmol e^−^ m^−2^ s^−1^)**	***E*_*k*_ (μmol photons m^−2^ s^−1^)**
Z802-LL	0.246 ± 0.004^Bb^	97.2 ± 0.8^Ab^	395.1^Ab^
Z802-HL	0.253 ± 0.004^Aa^	83.1 ± 0.7^Bb^	328.5^Bb^
*pgl*-LL	0.255 ± 0.003^Aa^	160.9 ± 1.3^Ba^	631.0^Ba^
*pgl*-HL	0.250 ± 0.003^Ba^	185.9 ± 2.1^Aa^	743.6^Aa^

*α, Photosynthetic efficiency measured by the initial slope of the RLC before the onset of saturation; rETR_max_, relative electron transport maximum; E_k_, minimum saturating irradiance. Letters A and B indicate significant differences (P < 0.05) between treatments in the same genotype, while a and b indicate significant differences (P < 0.05) between genotypes under the same treatment*.

Figure 6B shows the efficiency of photosystem II photochemistry, Φ_*PSII*_, in response to different actinic light. This parameter measures the proportion of light absorbed by chlorophyll associated with PSII and used in photochemistry, providing a measure of the rate of linear electron transport, and therefore, an indication of overall photosynthesis. Φ_*PSII*_ decreased with increasing PAR, and was higher in *pgl* than Z802 under all light intensities. Moreover, in *pgl*, PSII efficiency was generally higher under HL, unlike Z802, which was more efficient under LL. Photochemical (*qP*, Figure 6C) and non-photochemical quenching parameters (*NPQ*, Figure 6D) describe the relative effects of the energy dissipation pathway. *qP* gives an indication of the proportion of PSII reaction centers that are open, while *NPQ* measures energy flow into heat associated with xanthophyll cycle activity. Quenching coefficients plotted as a function of PAR showed a clear increase in *NPQ* and a steady decline in *qP* with increasing irradiance. *NPQ* in the Z802 genotype was nearly two times greater than that in *pgl* with increasing light. Moreover, the extent of decline in *qP* was considerably smaller in *pgl* than Z802 with increasing PAR. Overall, the RLCs and RLC-derived parameters suggest high photon-electron conversion efficiency and reduced non-photochemical quenching in *pgl*, thereby lowering energy losses.

### The size of light-harvesting chlorophyll antenna

**Figure 7:**
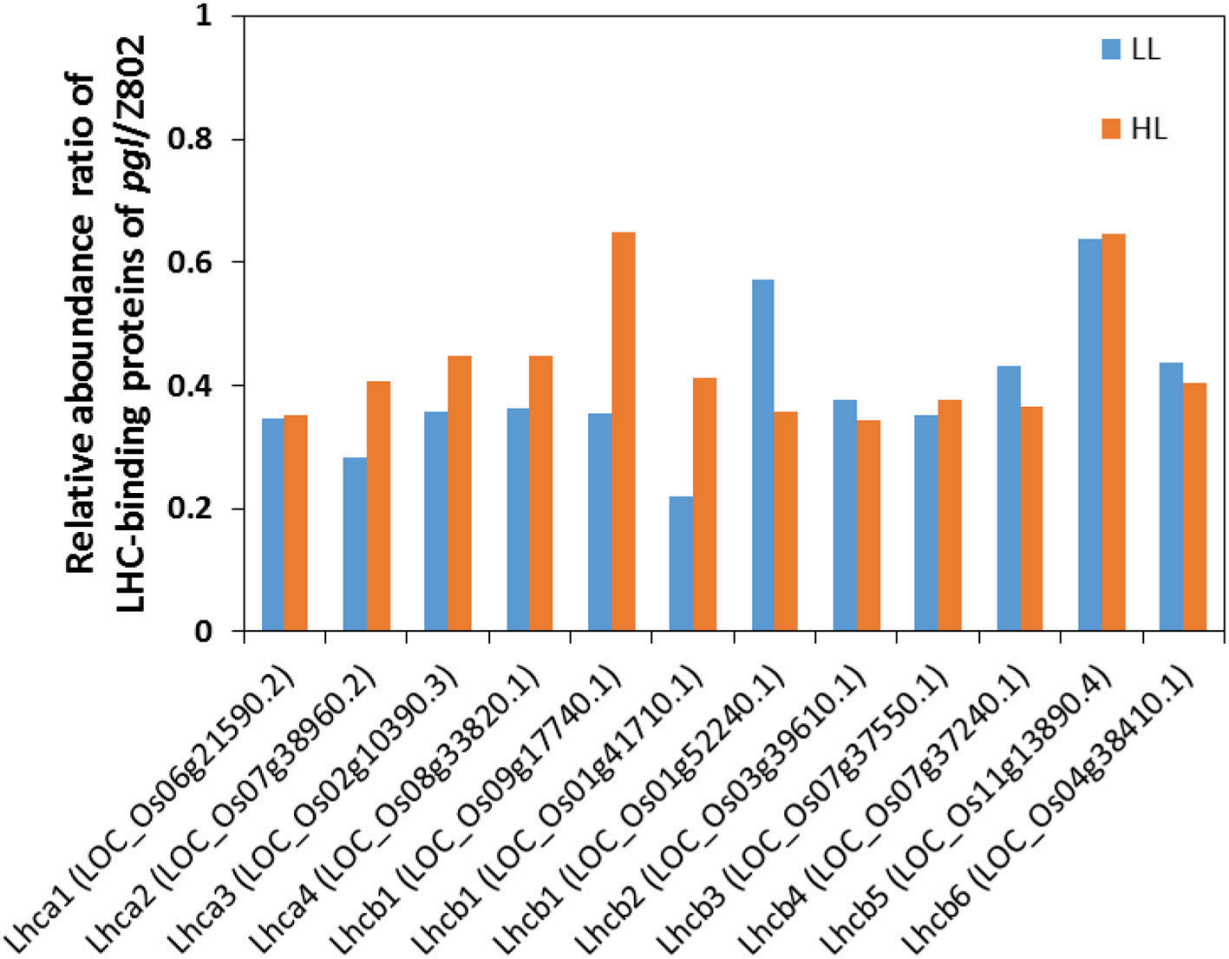
The relative abundance of ratio of light harvesting complex (LHC) binding proteins of *pgl*/Z802 in low light (LL) and high light (HL) treatments. The proteins of LHCI (Lhca1-4) and LHCII (Lhcb1-6) ligate chlorophylls and carotenes, which absorb light and transmit the excitation energy to the core complex.

Light energy is captured by large, discrete complexes of chlorophyll molecules bound to proteins and embedded in the thylakoid membranes. The majority of the chlorophyll molecules in a photosystem make up chlorophyll antenna. The antenna contains light-harvesting complexes. The relative abundance of LHC-binding proteins (Figure 7) and chlorophyll content (Figure 2) indicated a truncated light-harvesting chlorophyll antenna size in *pgl*. This is also confirmed by the higher Chl *a*/*b* in *pgl*, as chlorophyll *a* is tend to locate in the reaction center, while chlorophyll *b* is tend to locate in antenna. The binding proteins of LHC of photosystem I (LHCI) are Lhca1, Lhca2, Lhca3, and Lhca4. The binding proteins of LHC of photosystem II (LHCII) are Lhcb1, Lhcb2, Lhcb3, Lhcb4, Lhcb5, and Lhcb6 (Figure 7).

### Generation of ROS and photoinhibitory damage under high irradiance

**Figure 8:**
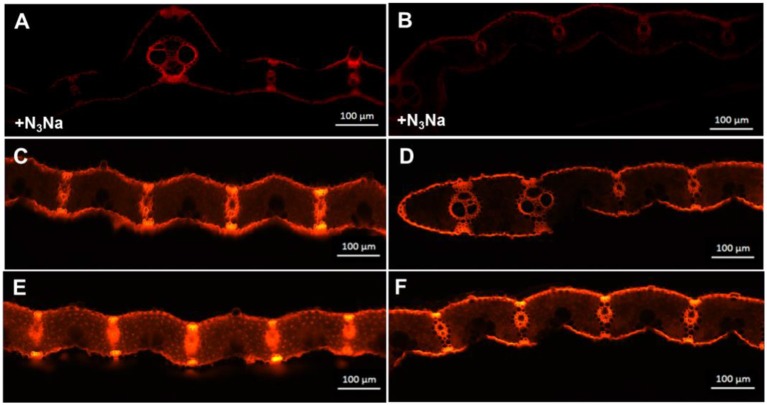
Imaging of reactive oxygen species (ROS) in leaves of Z802 and *pgl*. ${\text{O}}_{2}^{-}$-dependent DHE fluorescence in leaves of Z802 **(A,C,E)** and *pgl* **(B,D,F)**. **(A,B)** As a negative control, leaf samples were incubated with 1 mM N_3_Na (peroxidase inhibitor). **(C,D)** Leaves under low light (PPFD: ~200 μmol m^−2^ s^−1^) and **(E,F)** under high light (~1,000 μmol m^−2^ s^−1^). Bars = 100 μm.

**Figure 9:**
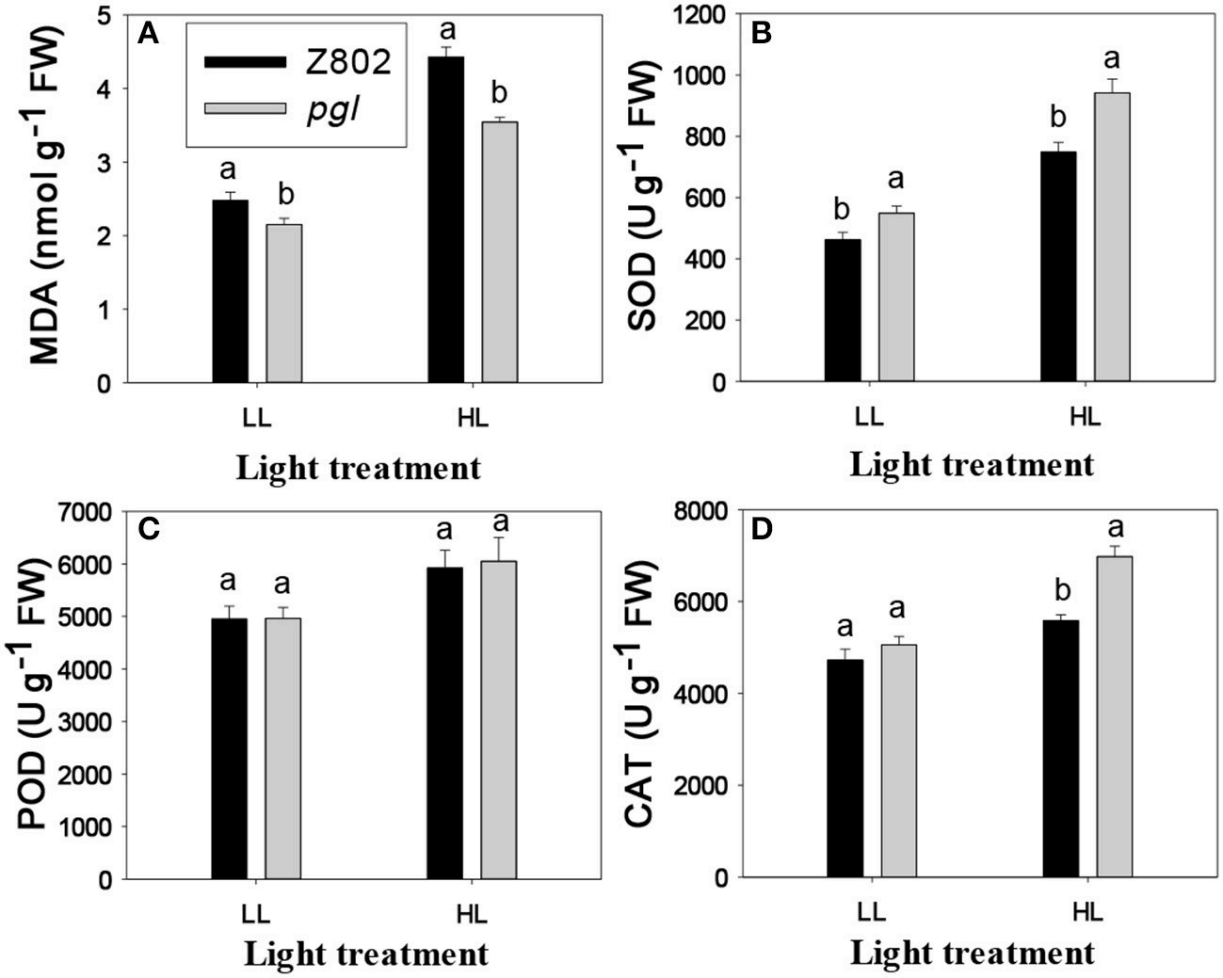
Lipid peroxidation (MDA, **A**), and antioxidant enzymes activity (superoxide dismutase, SOD, **B**; peroxidase, POD, **C**; catalase, CAT, **D**) in leaves of Z802 and *pgl* under low (LL) and high light treatment (HL). Different letters indicate significant differences (*P* < 0.05) between genotypes at LL or HL treatment.

*In vivo* detection of ROS in the leaves was carried out using a fluorescence microscope (Figure 8). To observe ${\text{O}}_{2}^{-}$ production, DHE, which is specific for this anion, was used as a probe (Zhao et al., 2003). DHE is also considered a marker of cell death, showing higher fluorescence signals in the zone around epidermal cells and vascular bundles (Rodríguez-Serrano et al., 2006; Figure 8). However, differences in fluorescence signals from mesophyll cells, indicating the location of chloroplasts, were found between the two genotypes (Figure 8). Fluorescence signal intensities under ZEN imaging were 95.0 and 69.5 for Z802 and *pgl* in the negative control (Figures 8A,B), respectively, compared with 242.7 and 149.1 for Z802 and *pgl* under LL (Figures 8C,D), and 455.2 and 283.0 for Z802 and *pgl* under HL, respectively (Figures 8E,F). These values combined with the ROS imaging data suggest that ${\text{O}}_{2}^{-}$ was depleted by N_3_Na in the negative control, but increased with increasing light intensity, especially in Z802 under HL. In general, ${\text{O}}_{2}^{-}$ radical accumulation was higher in Z802 compared to *pgl*, suggesting photoinhibitory damage in leaves of Z802. This is consistent with the content of MDA, a marker of lipid peroxidation (Figure 9). SOD, CAT, and POD are responsible for the detoxification of ${\text{O}}_{2}^{-}$ and H_2_O_2_, thereby preventing the formation of superoxide radicals. SOD, POD, and CAT activity increased in both Z802 and *pgl* under LL compared with HL. Activities of these enzymes suggest oxidative stresses under HL. Except for POD activity, both SOD and CAT activity were higher in *pgl* than Z802 under HL, possibly due to the photo-oxidative damage in Z802.

### Thermal imaging of the rice canopy and midday depression of photosynthesis

**Figure 10:**
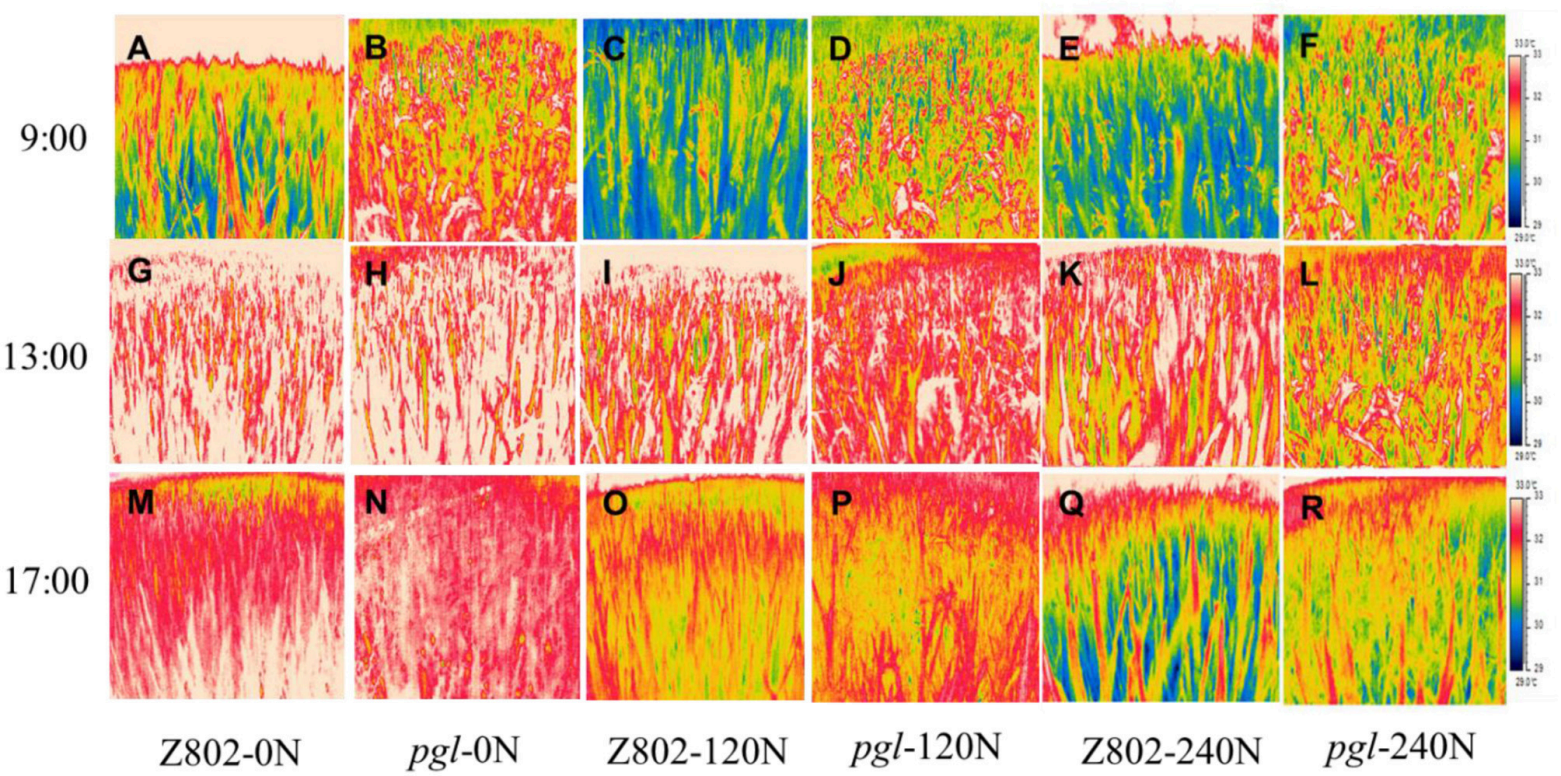
Thermal images of the canopy of Z802 and *pgl* in the morning (9:00), at noon (13:00) and in the afternoon (17:00) at the flowering stage under different rates of nitrogen (0, 120, 240 N).

**Figure 11:**
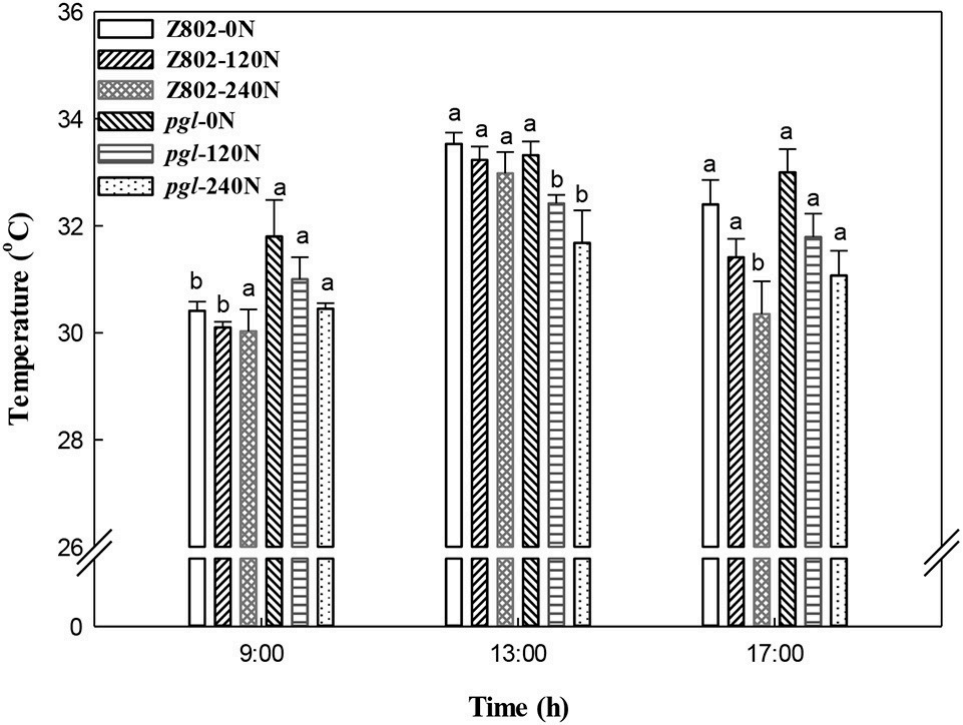
Canopy temperatures of Z802 and pgl in the morning (9:00), at noon (13:00) and in the afternoon (17:00) under different rates of nitrogen (0, 120, 240 N). Vertical bars denote standard deviations (*n* = 10). Different letters indicate significant differences (*P* < 0.05) between genotypes at the same level of N and at the same time of day.

As shown in Figures 10, 11, nitrogen fertilizer significantly reduced the canopy temperature. The average canopy temperature of Z802 decreased from 32.1°C at 0 N to 31.6 and 31.1°C at 120 and 240 N, respectively. In *pgl*, the average canopy temperature decreased from 32.7°C to 31.7 and 31.1°C, respectively (Figures 10, 11). The temperature of the canopy differed between the two rice genotypes, regardless of *N* inputs. The average canopy temperature was 0.9 and 0.6°C higher in *pgl* than Z802 in the morning (9:00) and afternoon (15:00), respectively, and 0.8°C lower at noon (13:00). The differences of canopy temperature at noon between genotype Z802 and *pgl* were related to the different chlorophyll contents in Z08 and *pgl*. The chlorophyll contents in Z802 were 2.45, 2.98, and 3.34 mg g^−1^ FW, at 0, 120, and 240 N, respectively, while the chlorophyll contents in *pgl* were 1.38, 1.47, and 1.67 mg g^−1^ FW, at 0, 120, and 240 N, respectively.

**Figure 12:**
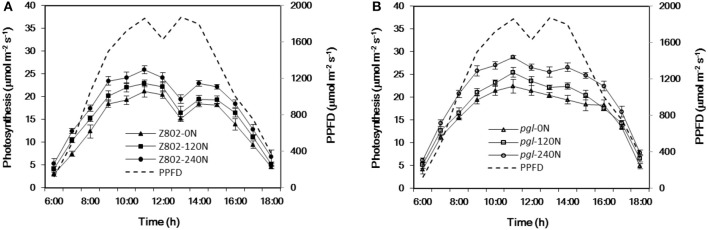
Diurnal variation in the net photosynthetic rates of Z802 **(A)** and *pgl* **(B)** under different rates of nitrogen (0, 120, 240 N) at the flowering stage. Data represent the mean of four replicates with the standard deviation shown by vertical bars.

The canopy temperature is strongly correlated with midday depression of photosynthesis. The average photosynthetic rate decreased by 27.1 and 11.0% when comparing net photosynthesis at 13:00 and at 11:00 for Z802 and *pgl*, respectively (Figure 12). A particularly large drop in photosynthesis at noon was observed in Z802. This phenomenon of midday depression of photosynthesis was also strongly correlated with the higher canopy temperature at noon in Z802.

## Discussion

Plants tend to overinvest in the synthesis of chlorophyll, assembling a large array of chlorophyll antennae. These antennae consist of a large number of protein-bound chlorophyll molecules that absorb photons, transferring their energy to the photosynthesis reaction center (Melis, 2009). However, only a few of these chlorophyll molecules are constituents of the actual reaction centers, most remaining in the antennae. Oversized chlorophyll antennae ensure a selective advantage, allowing interception and absorption of more light than neighboring competitors, even more than is actually needed (Ort et al., 2011, 2015). Prevailing sunlight intensities are also much higher than that required for saturation of photosynthesis, resulting in over-excitation of the photosystems, induction of NPQ, and a decrease in solar energy conversion efficiency. We therefore speculated that a genotype with low chlorophyll content could improve solar conversion efficiency in rice, mitigating the effects of photoinhibitory damage, and allowing adaptation to the sunny high-temperature environments where rice grows.

Figure 6A shows the rates of electron transport plotted as a function of photosynthetic active photons (μmol photons m^−2^ s^−1^). ETR linearly increased with increasing light intensity under a low light intensity range, suggesting that light absorption and light reactions, rather than carbon reactions, are limiting the photosystems (Gu et al., 2012a). Within this range of light intensities, photosynthesis operated at a maximum solar energy conversion efficiency. However, as light intensity increased, the slope of ETR decreased eventually approaching a plateau, the *r*ETR_max_ (Table 1). This shows that a higher intensity of sunlight and increased absorption of solar photons does not translate into photosynthetic electron transport. Photosynthesis limitations are imposed either by the Calvin-Benson cycle (slow catalytic rate of Rubisco), or the bottleneck of electron transport through the cytochrome *b*_6_/*f* complex in the thylakoid membranes, or relatively slow turnover rate of the Mn-containing H_2_O-oxidantion complex (Melis, 2009; Foyer and Shigeoka, 2011). As shown in Figure 6A, saturation of photosynthesis (*E*_*k*_) in Z802 was reached at 395.1 μmol photons m^−2^ s^−1^ under LL and at 328.5 μmol m^−2^ s^−1^ under HL, and in *pgl* at 631.0 and 743.6 μmol m^−2^ s^−1^, respectively. Taking into account the fact that sunlight intensities can reach up to 2,500 μmol m^−2^ s^−1^, these findings suggest that ~85.5 and 72.5% of the solar energy is wasted in Z802 and *pgl*, respectively, under high irradiance. The average *r*ETR_max_ of *pgl* was 173.4 μmol e^−^ m^−2^ s^−1^, almost twice that of Z802 (Table 1), suggesting a higher capacity for photosynthesis. This is consistent with previous findings whereby higher photosynthetic activity was reported with low chlorophyll content in chlorophyll-deficient mutants of various plant species (Edwards et al., 1993; Habash et al., 1994; Havaux and Tardy, 1997; Li et al., 2013; Jin et al., 2016; Kirst et al., 2017; Slattery et al., 2017), algae (Melis et al., 1998; Polle et al., 2003), and cyanobacteria (Kirst et al., 2014).

Photosynthesis is an important source of cellular oxidants (Foyer and Shigeoka, 2011), with formation of triple chlorophylls (Hideg et al., 1998). By reacting with ground-state triplet oxygen, triple chlorophylls generate harmful singlet oxygen, which can cause cellular injury. Under moderate stress, plants appear to purposefully generate ROS as signaling molecules to control processes such as pathogen defense responses, programmed cell death, and stomatal behavior (Apel and Hirt, 2004; Pospíšil, 2016). However, when the energy absorbed by the photosystems exceeds the level that can be used by carbon fixation reactions and the scavenging system is unable to sufficiently eliminate undesirable ROS formation, oxidative damage of proteins, DNA, and lipids can occur (Apel and Hirt, 2004), as seen in Z802. For example, herbicides that inhibit the synthesis of carotenoids cause the production of vast amounts of ROS, which cause the chlorophyll to bleach and subsequently kill the plant (Wakabayashi and Böger, 2002). ROS inhibit the repair of PSII, in particular the synthesis of D1 protein, at the mRNA translation level (Nishiyama et al., 2006). As shown in Figure 8, more ROS accumulated in Z802 than *pgl* under HL, while as shown in Figure 4, more osmophilic bodies were observed in Z802 than *pgl* under HL. These findings suggest disruption of granum thylakoids and possible damage to the chloroplasts in Z802. Moreover, MDA, a sign of lipid peroxidation, was also higher in Z802 (Figure 9). Combined, these data suggest photoinhibitory damage in Z802, especially under HL. Leaves with low chlorophyll (i.e., *pgl*) produce less singlet oxygen under strong light compared to those with higher amounts of chlorophyll (Z802). Consequently, a pale leaf genotype is less prone to photo-oxidative damage of thylakoid membrane lipids and/or proteins (Tardy et al., 1998). This also explains why the two genotypes showed clearly distinctive patterns of chlorophyll content (Figure 2), light absorption (Figure 5), and RLC (Figure 6). Thus, in general, chloroplast development and photosynthesis were improved in the *pgl* genotype under HL, while an opposite trend was found in Z802.

Under natural conditions of high temperature and intense sunlight, two net photosynthetic peaks occur, one in late morning and another in late afternoon, with a depression around noon, the so-called midday depression of photosynthesis (Figure 12). Excess light, in combination with a high leaf temperature, is the major environmental stress causing midday depression of photosynthesis (Valladares and Pearcy, 1997; Muraoka et al., 2000). Gu et al. (2012b) also found temperature to be a major factor limiting factor of photosynthesis under field conditions. Maintenance of a “low” leaf temperature at noon could therefore have important advantages in sunny environments, since photoinhibition of photosynthesis is temperature dependent, with elevated temperatures strongly exacerbating photoinhibition (Tardy et al., 1998).

In our experiment, the application of N fertilizer significantly decreased the canopy temperature as shown in Figure 11 and consistent with a previous study (Seligman et al., 1983). Maintaining a low concentration of chlorophyll in the leaf tissue is a general feature of plants that have genetically adapted to harsh environments (Maslova and Popova, 1993; Kyparissis et al., 1995; Tardy et al., 1998), which have been noted as the likely photoprotection mechanism mitigating the damaging effect of leaf heating in wild grasses and cereal landraces adapted to semi-arid environments (Havaux and Tardy, 1999; Zaharieva et al., 2001; Royo et al., 2014; Ruíz et al., 2016). Similarly, in this study, the *pgl* genotype with its lower chlorophyll content was also more resistant to the midday depression in photosynthesis (Figure 12) due to its cooler canopy (Figure 11).

## Conclusion

Plants tend to overinvest in chlorophyll synthesis, with assembly of large chlorophyll antenna molecules in their photosynthetic apparatus. These large antennae absorb excess light energy relative to the capacity of the photosystems. This excess light energy subsequently results in the generation of high levels of ROS or is dissipated as heat by *NPQ*. It is therefore a waste of energy, thereby decreasing the solar energy conversion efficiency. In this study, however, the *pgl* genotype with truncated light-harvesting chlorophyll antenna showed a low level of *NPQ* and ROS generation. Photosystem II efficiency and ETR were also high in the *pgl* genotype. Combined, these data indicate that selecting of a rice genotype with a low chlorophyll content could improve solar conversion efficiency, mitigating the effects of photoinhibitory damage and improving adaptation to the sunny high-temperature environments where rice grows. Although, the physiological mechanisms were discussed, the genetic basis remains obscure and requires further studies. Furthermore, physiological studies, especially quantitative proteomics studies should combined with modeling analyses (Friend, 1991; Hikosaka and Terashima, 1995; Medlyn, 1996; Hikosaka, 1997, 2005; Xu et al., 2012) to reveal how large and what ratio of light harvesting complexes among the photosystems are optimal for the efficiency of photosynthesis.

## Author contributions

JG, JY designed the research. JG, ZZ, ZL, YC, ZW, and HZ performed research. JG, ZZ, and JY analyzed the data. JG, ZZ, and JY wrote the paper.

### Conflict of interest statement

The authors declare that the research was conducted in the absence of any commercial or financial relationships that could be construed as a potential conflict of interest. The reviewer HM and handling Editor declared their shared affiliation, and the handling Editor states that the process met the standards of a fair and objective review.
