# Supplementary material for: Photosynthetic Properties and Potentials for Improvement of Photosynthesis in Pale Green Leaf Rice under High Light Conditions
Source: Front Plant Sci. 2017 Jun 20;8:1082. doi: 10.3389/fpls.2017.01082 (PMC5476740; doi:10.3389/fpls.2017.01082)
Supplement: Supplementary file 1 [file DataSheet1.docx]

**Supplementary Table S1**

Table S1. Yield, yield components, and biomass of Z802 and *pgl* for the field experiment under different rates of nitrogen (0 N, 120 N, 240 N)

| N rates | Genotype | PN | SPP | GR | GW | D | Y | Y/D | B | B/D |
| --- | --- | --- | --- | --- | --- | --- | --- | --- | --- | --- |
| (kg N ha^-1^) |  | (×10^6^ ha^-1^) |  | (%) | (g) | (d) | (t ha^-1^) | (kg ha^-1^ d^-1^) | (t ha^-1^) | (kg ha^-1^ d^-1^) |
| 0 | Z802 | 1.74a | 120b | 90.91a | 24.08a | 133a | 4.27a | **32.11a** | 9.74a | **73.26b** |
|  | *pgl* | 1.56b | 138a | 78.63b | 24.37a | 124b | 3.91b | **31.53a** | 9.34a | **75.32a** |
| 120 | Z802 | 2.25a | 126a | 88.33a | 24.42a | 131a | 5.94a | **45.34b** | 12.92a | **98.63b** |
|  | *pgl* | 2.13b | 126a | 81.21b | 24.47a | 123b | 5.74a | **46.67a** | 13.60a | **110.57a** |
| 240 | Z802 | 2.58a | 124b | 87.62a | 24.81a | 130a | 6.79a | **52.23b** | 14.84a | **114.15b** |
|  | *pgl* | 2.52a | 132a | 80.40b | 25.05a | 123b | 6.56a | **53.33a** | 15.27a | **124.15a** |

PN, panicle number per area (×10^6^ ha^-1^); SPP, spikelets per panicle; GR, grain-filling ratio (%); GW, 1000-grain weight (g); D, growth duration; Y, yield (t ha^-1^); B, biomass (t ha^-1^) which was determined from seed sowing (d). Significant differences (P< 0.05) between YL and WT in each treatment were indicated by different letters.
